# New Appliances and Things Medical

**Published:** 1902-06-21

**Authors:** 


### NEW APPLIANCES AND THINGS MEDICAL.

[We shall be glad to receive at our Office, 28 & 29 Southampton Street, Strand, London, W.C., from the manufacturers, specimens of all new preparations and appliances which may be brought out from time to time.]

### SPONGIO PILINE.

(R. R. Whitehead and Brothers, Limited, 10 Endell Street, Long Acre, London, W.C.)

Spongio piline, which was the invention of the late Dr. Markwick, has long been held by the medical profession as one of the most useful of media for the application of heat to the surface of the body. It consists of a specially prepared and compressed tissue, backed with a resistant material, which retains the heat, and which is to a considerable extent waterproof. For fomenting, and for the application of stupes it is infinitely superior to the old method of using lint and covering with oil silk. Spongio piline subserves the double function of these two materials, and at the same time retains the heat in a more efficient manner and more agreeably to the patient.

### THERMOFUGE.

(Parke, Davis and Co., 11 Queen Victoria Street, London, E.C.)

This preparation is quite original in conception. It may be regarded as a calorific and anodyne paste, which should be spread on the affected part like butter, and covered by a simple dressing or by spongio piline. The preparation contains aluminum silicate, glycerine, boric acid, menthol, thymol, etc., and by its constitution will at once suggest to the medical mind its convenient substitution for poultices, stupes, or anodyne plasters. Practically this new paste is found to be comfortable to the patient, and very efficacious for the treatment of lumbago, sciatica, pleurodynia, pleurisy, etc.; it will probably be largely used for these purposes when its merits as a detergent antiseptic application are more generally known.

### THE SENTRY FILTER.

(Barnett and Foster, Niagara Works, Eagle Wharf Road, London, N.)

The "Sentry," which is the registered trade mark of a series of filters manufactured by Barnett and Foster, applies to a system of filtering the general plan of which is based on the Pasteur model, although the material used is different, and which is equally satisfactory whether employed on a large or on a small scale. For domestic use the "Sentry" table filter consists of a lower glass jar, which holds about $1\frac{3}{4}$ pints, surmounted by a glass cylinder containing a filter tube of insoluble stone. The water to be filtered passes through from the outside to the inside, and deposits all suspended matter on the outer surface, whence it may be removed by occasional washing. These tubes, though thicker and stronger than those usually employed for this purpose, permit of a rapid filtration. Modified tubes with fittings can be applied to service pipes. This method, to our thinking, is the most reliable for the filtration of drinking-water. If fitted to a tap directly off the main, the water is free from the contamination which is inseparably associated with water which has passed through a cistern. The method of applying the "Sentry" Service filter is extremely simple, and the cost only 16s. "Sentry" filters can also be supplied mounted with pumps, where a pressure service does not exist. Camp filters of all kinds, as well as those for pocket and travelling use, are made on the same satisfactory principle. We regard these filters as thoroughly practical and scientific, and, considering their substantial make, exceedingly cheap and economical.

